# Differences in the clinical and hormonal presentation of patients with familial and sporadic primary aldosteronism

**DOI:** 10.3389/fendo.2024.1336306

**Published:** 2024-03-01

**Authors:** Marta Araujo-Castro, Paola Parra, Patricia Martín Rojas-Marcos, Miguel Paja Fano, Marga González Boillos, Eider Pascual-Corrales, Ana María García Cano, Jorge Gabriel Ruiz-Sanchez, Almudena Vicente Delgado, Emilia Gómez Hoyos, Rui Ferreira, Iñigo García Sanz, Mònica Recasens Sala, Rebeca Barahona San Millan, María José Picón César, Patricia Díaz Guardiola, Carolina M. Perdomo, Laura Manjón-Miguélez, Rogelio García Centeno, Ángel Rebollo Román, Paola Gracia Gimeno, Cristina Robles Lázaro, Manuel Morales-Ruiz, María Calatayud, Simone Andree Furio Collao, Diego Meneses, Miguel Sampedro Nuñez, Verónica Escudero Quesada, Elena Mena Ribas, Alicia Sanmartín Sánchez, Cesar Gonzalvo Diaz, Cristina Lamas, María del Castillo Tous, Joaquín Serrano Gotarredona, Theodora Michalopoulou Alevras, Eva María Moya Mateo, Felicia A. Hanzu

**Affiliations:** ^1^ Endocrinology and Nutrition Department, Hospital Universitario Ramón y Cajal, Madrid, Spain; ^2^ Instituto de Investigación Biomédica Ramón y Cajal (IRYCIS), Madrid, Spain; ^3^ Endocrinology and Nutrition Department, Hospital Universitario La Paz, Madrid, Spain; ^4^ Endocrinology and Nutrition Department, OSI Bilbao-Basurto, Hospital Universitario de Basurto, Bilbao, Spain; ^5^ Medicine Department, Basque Country University, Bilbao, Spain; ^6^ Endocrinology and Nutrition Department, Hospital Universitario de Castellón, Castellón, Spain; ^7^ Biochemistry Department, Hospital Universitario Ramón y Cajal, Madrid, Spain; ^8^ Endocrinology and Nutrition Department, Hospital Universitario Fundación Jiménez Díaz, Madrid, Spain; ^9^ Endocrinology and Nutrition Department, Hospital Universitario de Toledo, Toledo, Spain; ^10^ Endocrinology and Nutrition Department, Hospital Universitario de Valladolid, Valladolid, Spain; ^11^ Endocrinology and Nutrition Department, Hospital Universitario Rey Juan Carlos, Madrid, Spain; ^12^ General and Digestive Surgery Department, Hospital Universitario de La Princesa, Madrid, Spain; ^13^ Endocrinology and Nutrition Department, Hospital De Girona Doctor Josep Trueta, Girona, Spain; ^14^ Endocrinology and Nutrition Department, Hospital Universitario Virgen de la Victoria de Málaga, IBIMA, Malaga, Spain; ^15^ CIBEROBN, Madrid, Spain; ^16^ Endocrinology and Nutrition Department, Hospital Universitario Infanta Sofía, Madrid, Spain; ^17^ Endocrinology and Nutrition Department, Clínica Universidad de Navarra, Pamplona, Spain; ^18^ Endocrinology and Nutrition Department, Hospital Universitario Central de Asturias, Oviedo, Spain; ^19^ Instituto de Investigación Sanitaria del Principado de Asturias (ISPA), Oviedo, Spain; ^20^ Endocrinology and Nutrition Department, Hospital Universitario Gregorio Marañón, Madrid, Spain; ^21^ Endocrinology and Nutrition Department, Hospital Reina Sofía, Córdoba, Spain; ^22^ Endocrinology and Nutrition Department, Hospital Royo Villanova, Zaragoza, Spain; ^23^ Endocrinology and Nutrition Department, Complejo Universitario de Salamanca, Salamanca, Spain; ^24^ Biochemistry and Molecular Genetics Department-CDB, Hospital Clinic, IDIBAPS, CIBERehd, Barcelona, Spain; ^25^ Endocrinology and Nutrition Department, Hospital Doce de Octubre, Madrid, Spain; ^26^ Endocrinology and Nutrition Department, Hospital Universitario La Princesa, Madrid, Spain; ^27^ Nephrology Department, Hospital Universitario Doctor Peset, Valencia, Spain; ^28^ Endocrinology and Nutrition Department, Hospital Universitario Son Espases, Palma de Mallorca, Spain; ^29^ Endocrinology and Nutrition Department, Hospital Universitario De Albacete, Albacete, Spain; ^30^ Endocrinology and Nutrition Department, Hospital Universitario Virgen Macarena, Sevilla, Spain; ^31^ Endocrinology and Nutrition Department, Hospital General Universitario de Alicante, Alicante, Spain; ^32^ Endocrinology and Nutrition Department, Hospital Joan XXIII, Tarragona, Spain; ^33^ Internal Medicine, Hospital Universitario Infanta Leonor, Madrid, Spain; ^34^ Endocrinology and Nutrition Department, Hospital Clinic, IDIPAS, Barcelona, Spain

**Keywords:** primary aldosteronism, familial hyperaldosteronism, genetic study, pathogenic variant, plasma aldosterone concentration

## Abstract

**Purpose:**

To compare the clinical and hormonal characteristics of patients with familial hyperaldosteronism (FH) and sporadic primary aldosteronism (PA).

**Methods:**

A systematic review of the literature was performed for the identification of FH patients. The SPAIN-ALDO registry cohort of patients with no suspicion of FH was chosen as the comparator group (sporadic group).

**Results:**

A total of 360 FH (246 FH type I, 73 type II, 29 type III, and 12 type IV) cases and 830 sporadic PA patients were included. Patients with FH-I were younger than sporadic cases, and women were more commonly affected (P = 0.003). In addition, the plasma aldosterone concentration (PAC) was lower, plasma renin activity (PRA) higher, and hypokalemia (P < 0.001) less frequent than in sporadic cases. Except for a younger age (P < 0.001) and higher diastolic blood pressure (P = 0.006), the clinical and hormonal profiles of FH-II and sporadic cases were similar. FH-III had a distinct phenotype, with higher PAC and higher frequency of hypokalemia (P < 0.001), and presented 45 years before sporadic cases. Nevertheless, the clinical and hormonal phenotypes of FH-IV and sporadic cases were similar, with the former being younger and having lower serum potassium levels.

**Conclusion:**

In addition to being younger and having a family history of PA, FH-I and III share other typical characteristics. In this regard, FH-I is characterized by a low prevalence of hypokalemia and FH-III by a severe aldosterone excess causing hypokalemia in more than 85% of patients. The clinical and hormonal phenotype of type II and IV is similar to the sporadic cases.

## 1 Introduction

Primary aldosteronism (PA) is the most common cause of secondary hypertension, accounting for 10% of hypertensive patients in the general setting and of 20% in patients with refractory hypertension (1). Approximately 95% of these cases are sporadic, with the remaining 5% caused by a pathogenic variant in genes that result in an increase in the transcription and expression of CYP11B2, which is responsible for aldosterone synthesis (familial hyperaldosteronism (FH) types II, III, and IV) or a fusion of the *CYP11B2* and *CYP11B1* genes, which is responsible for FH-I or glucocorticoid-remediable PA (GRA) (2).

Sutherland et al. described the familial occurrence of PA for the first time in 1966 (3). Lifton et al. (4) demonstrated the genetic basis of this familial form: GRA caused by a hybrid gene composed of ACTH-regulated 11b-hydroxylase (CYP11B1) regulatory sequences and aldosterone synthase (CYP11B2) coding sequences. Following that, a number of FH type I cases have been described in the literature (5–29). Gordon et al. (30) described the first case of FH type II several years after the discovery of FH-I. Since its first description, more than 80 cases have been reported in the literature (6, 31–41). More recently, two additional kinds of genetic PA have been identified: type III caused by a pathogenic mutation in *KCNJ5* (42) and type IV caused by a *CACNA1H* pathogenic variant (43).

However, few studies have directly compared the clinical and hormonal aspects of the familial and sporadic PA cases (11, 33), despite the well-known description of the familial cases. Michael Stowasser (33) was the first to compare 88 consecutive PA patients and 13 FH type II patients. There were no differences in age at presentation, sex incidence, and biochemical parameters between both groups. Another series comparing family members with positive (n = 21) and negative (n = 18) study for FH-I found that body mass index (BMI) was lower and plasma aldosterone concentration (PAC) higher in FH-I; however, the control group did not have PA in this study (26). This study design was comparable with the Litchfield study (25). A subsequent study matched 79 GRA-positive patients and 114 GRA-negative unilateral PA patients by age, gender, and BMI. They found that being younger and having a lower PAC were associated with a higher probability of FH-I (11). Nevertheless, the majority of these studies contained a small number of patients and only evaluated the differences between sporadic PA cases and FH type I and type II cases.

Considering this background, the aim of our study was to identify the clinical and hormonal features linked to FH by comparing a large cohort of FH patients to a group of sporadic PA cases. Based on that information, we will aim to determine which PA patients should be genetically tested.

## 2 Methods

### 2.1 Patients

The control group of sporadic cases was extracted from the Spanish Primary Aldosteronism (SPAIN-ALDO) Registry of the Spanish Endocrinology and Nutrition Society (SEEN). As previously described (44), this is a multicenter collaborative study involving patients with a diagnosis of PA who were followed up in 35 Spanish tertiary hospitals between January 2018 and July 2023. At the time of data analysis (11/08/2023), the registry contained 855 patients with PA.

**Figure 1:**
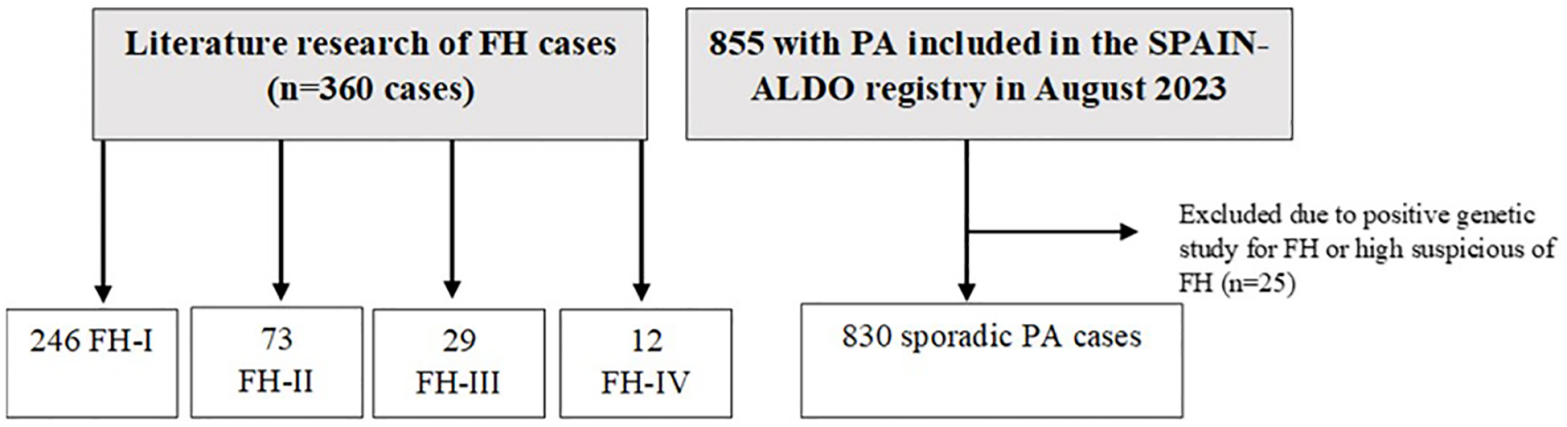
Study population. PA, primary aldosteronism; FH, familial hyperaldosteronism.

We excluded the following patients from the sporadic group: (i) those patients with a positive genetic study (n = 1); (ii) those with familial history of PA and no available negative genetic study for FH (n = 13); (iii) patients who underwent genetic testing due to high suspicion and whose results were pending (n = 7); and (iv) patients diagnosed of PA before the age of 30 and no available negative genetic study for FH (n = 4). Thus, 830 cases were included. Of these 830 patients, only 18 underwent genetic study for FH, with negative results (**Figure 1**).

For the diagnosis of FH, we followed the following criteria: Diagnosis of FH-I was performed by long-PCR amplification of the hybrid gene (*CYP11B1/CYP11B2*); diagnosis of FH-II was made when a mutation in *CLCN2* was detected or when there was a family history of PA in at least two family members; diagnosis of FH-III when a pathogenic variant in *KCNJ5* was demonstrated and FH-IV if a pathogenic variant in *CACNA1H* was detected.

### 2.2 Definitions

PA diagnosis was made following the recommendations of the clinical international guidelines for PA (45, 46). Blood samples for plasma renin activity (PRA) and/or concentration (PRC) and PAC were collected from all patients. As we have previously described (47), those patients (n = 340) who did not meet the criteria of overt PA (hypokalemia, PAC >18 ng/dL and pathological aldosterone-to-renin ratio) underwent at least one of the following confirmatory tests: oral sodium loading, saline infusion test, captopril challenge test, and/or fludrocortisone suppression test.

The findings of the adrenal venous sampling (AVS) and/or the outcomes after adrenalectomy were used to differentiate between unilateral and bilateral PA. A total of 326 out of 830 patients underwent AVS, with 190 being successful. Unilateral disease was assumed if the lateralization index of the aldosterone to cortisol ratio was ≥4.0 on the dominant vs. non-dominant side during ACTH stimulation or at least two times higher under unstimulated conditions (1, 6). In patients without successful AVS, unilateral disease was assumed if a complete biochemical cure after surgery was obtained (n = 186). The PASO classification criteria were used to define biochemical and clinical cure for PA after adrenalectomy (48).

### 2.3 Search strategy for the identification of FH cases

**Figure 2:**
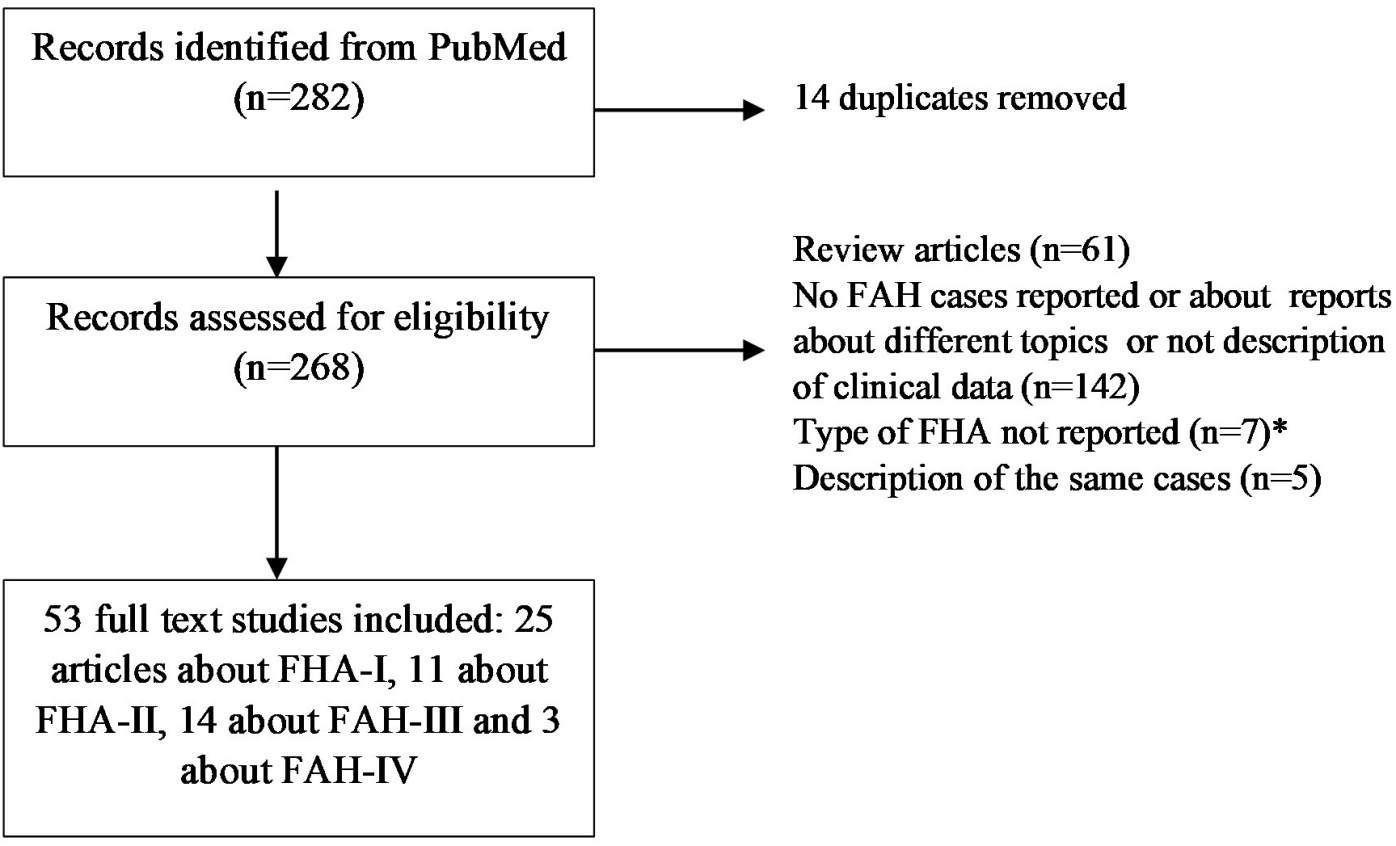
Flow-chart for identification of FAH cases. FH, familial hyperaldosteronism.

The FH cases were identified using the SANRA scale (49). The search strategy was conducted in PubMed without a date filter until 12/08/2023. The following keywords were used in the search: familial primary aldosteronism [TI]: 136 results; familial hyperaldosteronism [TI]: 61 results; hereditary aldosteronism [title]: 18 results; inherited primary aldosteronism [TI]: 16 results; dexamethasone-suppressible aldosteronism: 2 results; and glucocorticoid-remediable aldosteronism [TI]: 49 results. Potentially relevant articles were retrieved after reading the title, abstract, or whole article, and we discarded repeated articles. Only articles written in English were considered. The articles found through these searches as well as the pertinent references listed in those papers were reviewed. After that, 53 original articles were included: 25 articles about FH-I (5–29), 12 about FH-II (6, 31–41), 13 about FAH-III (32, 42, 50–60), and 3 about FAH-IV (43, 61, 62) (**Figure 2**).

### 2.4 Statistical analysis

All statistical analyses were conducted with STATA.15. Shapiro–Wilk’s test was used to assess the normality of continuous variables. All data are expressed as the mean and standard deviation for normally distributed variables and the median (and range) for non-normally distributed variables. Student’s t-test was used to compare quantitative variables and the *X* ^2^ test for qualitative variables between two groups. In all cases, a two-tailed P value < 0.05 was considered statistically significant.

## 3 Results

### 3.1 Differences between FH-I and sporadic cases

A total of 246 patients with confirmed FH-I were compared with 830 cases of sporadic PA. Patients with FH-I were found to be younger than sporadic cases, and women were more commonly affected than men. On the other hand, sporadic cases had higher PAC and lower PRA than FH-I. Besides, hypokalemia was uncommon (12%) in FH-I patients, but it reached a prevalence of 60% in sporadic cases. 40.3% of the FH-I patients (n = 77/129) were normotensive. No other differences were detected between the two groups (**Table 1**).

**Table 1:** Differences between FH-I and sporadic PA cases.

Variable	FH-I (n = 246)	Sporadic PA (n = 830)	P value
***Age, years***	33.6 ± 18.07	56.5 ± 4.76	<0.001
***% women***	51.9% (n = 126)	41.3% (n = 342)	0.003
***Systolic blood pressure, mmHg***	151.0 ± 20.05	150.3 ± 21.86	0.689
***Diastolic blood pressure, mmHg***	92.5 ± 10.44	89.7 ± 13.52	0.007
***Hypokalemia, %***	11.6% (n = 17/146)	59.6% (n = 486/816)	<0.001
***Serum K levels (mEq/L)***	3.9 ± 0.50	3.8 ± 1.67	0.304
***PAC (ng/dL)***	29.5 ± 15.03	44.4 ± 78.85	0.005
***PRA (ng/mL/h)***	1.3 ± 6.81	0.4 ± 0.86	0.004

FH, familiar hyperaldosteronism; PA, primary aldosteronism; PAC, plasma aldosterone concentration; PRA, plasma renin activity. Normal ranges for serum K levels: 3.5 mEq/mL–4.5 mEq/mL.

### 3.2 Differences between FH-II and sporadic cases

We compared 73 FH-II patients to 830 sporadic PA patients. The clinical and hormonal profiles were similar, except for a younger age and higher diastolic blood pressure in the group of FH-II (**Table 2**).

**Table 2:** Differences between FH-II and sporadic PA cases.

Variable	FH-II (n = 73)**	Sporadic PA (n = 830)	P value
**Age, years**	33.6 ± 19.65	56.5 ± 4.76	<0.001
**% women**	51.4% (n = 37)	41.3% (n = 342)	0.097
**Systolic blood pressure, mmHg**	154.6 ± 31.05	150.3 ± 21.86	0.230
**Diastolic blood pressure, mmHg**	97.3 ± 21.16	89.7 ± 13.52	0.006
**Hypokalemia, %**	53.9% (n = 35/65)	59.6% (n = 486/816)	0.373
**Serum K levels (mEq/L)**	3.5 ± 0.56	3.8 ± 1.67	0.164
**PAC (ng/dL)**	43.3 ± 45.4	44.4 ± 78.85	0.226
**PRA (ng/mL/h)**	0.82 ± 1.40	0.4 ± 0.86	0.017

FH, familiar hyperaldosteronism; PA, primary aldosteronism; PAC, plasma aldosterone concentration; PRA, plasma renin activity. Normal ranges for serum K levels: 3.5 mEq/mL–4.5 mEq/mL.

**FH-II was genetically demonstrated in 18 out of the 73 patients (pathogenic variant in the *CCLN2* gene); in the other 55 patients, the diagnosed was based on the presence of ≥2 members of the same family with confirmed PA (6).

### 3.3 Differences between FH-III and IV and sporadic cases

The 29 cases of FH type III and the 12 cases of FH-IV were compared with the sporadic cases. FH-III showed a distinct phenotype, with higher PAC, lower PRA, lower serum potassium levels, and a younger age at PA diagnosis, presenting 45 years earlier than the sporadic cases (**Table 3**). Due to problems in achieving proper blood pressure control, 17 of the 29 patients with FH-III underwent bilateral adrenalectomy.

**Table 3:** Differences between FH-III and IV and sporadic PA cases.

Variable	FH-III (n = 29)	FH-IV (n = 12)	Sporadic PA (n = 830)
**Age, years**	7.42 ± 11.51*	23.3 ± 20.41*	56.5 ± 4.76
**% women**	75.9% (n = 7)*	33.3% (n = 4)	41.3% (n = 342)
**Systolic blood pressure, mmHg**	154.4 ± 27.85	151.6 ± 20.08	150.3 ± 21.86
**Diastolic blood pressure, mmHg**	99.4 ± 19.36*	98.8 ± 17.56	89.7 ± 13.52
**Hypokalemia, %**	89.3% (n = 25/28)*	58.3% (n = 7/12)	59.6% (n = 486/816)
**Serum K levels (mEq/L)**	2.6 ± 0.74*	3.1 ± 0.71*	3.8 ± 1.67
**PAC (ng/dL)**	101.5 ± 80.73*	44.1 ± 31.22	44.4 ± 78.85
**PRA (ng/mL/h)**	0.18 ± 1.40*	1.3 ± 1.31*	0.4 ± 0.86

FH, familiar hyperaldosteronism; PA, primary aldosteronism; PAC, plasma aldosterone concentration; PRA, plasma renin activity. Normal ranges for serum K levels: 3.5 mEq/mL–4.5 mEq/mL.

* symbol refers to statistically significant differences between FAH cases when comparing with sporadic cases.

The clinical and hormonal phenotypes of FH type IV and sporadic cases were similar, except for lower age and serum potassium levels and higher PRA at presentation in the former (**Table 3**).

## 4 Discussion

The most consistent finding in FH patients was the younger age of the four types when compared with sporadic PA cases. Because FH is transmitted by autosomal dominant inheritance, the history of other members of the family with PA is another key indicator to infer hereditary PA (63). In fact, the current general recommendation for screening hereditary PA is for patients with early onset and a positive family history of PA (64).

When we compared FH type I and sporadic cases, we found that, in addition to being younger, FH-I had more severe hypertension than in sporadic PA patients. In this regard, it has been previously reported that severe hypertension in infancy and early adulthood is the most typical presentation of GRA (5). Nevertheless, some cases are just moderately hypertensive, if at all (65). According to this, we found that 40% of the FH-I patients had normal blood pressure levels and were classed as normotensive. However, even in normotensive FH-I patients, the aldosterone excess is associated with increased left ventricular wall thickness and reduced diastolic function when compared with normotensive subjects without GRA (23). Another notable feature of FH-I cases in our study was the low prevalence of hypokalemia, which occurred in less than 12% of the cases. Accordingly, a prevalence of 13% was described in the PATOGEN study (6) and in the Aglony et al. series (5). The real cause of hypokalemia in individuals with PA is due to the fact that the *CYP11B1/CYP11B2* hybrid gene is unknown; however, it may be connected to the fact that aldosterone secretion is regulated by ACTH instead of angiotensin II. Thus, the mineralocorticoid effect may be expected to be reduced. This hypothesis is supported by the observation that PRA was less suppressed in FH-I than in sporadic cases. Considering our results, it is important to take into account that FH-I may be hypokalemic or normokalemic. Thus, the presence of normal serum potassium levels does not discount genetic testing particularly in families where the penetrance of hypertension is inevitably variable.

In relation to FH type II, we found that the clinical and hormonal profile was comparable with sporadic cases, apart from a younger age and slightly higher diastolic blood pressure in the group of FH-II. Type II FH is caused by a pathogenic mutation in the *CLCN2* gene, leading to elevated intracellular Ca^2+^ concentration, which triggers depolarization and aldosterone secretion (43). The only previous study that compared sporadic and FH-II cases described that both groups had similar potassium levels and blood pressure levels. In addition, since the prevalence of aldosterone producing adenomas was not significantly different in FH-II and sporadic PA patients, the former group may have had radiological features similar to sporadic cases (6). Considering that the clinical picture does not allow to differentiate it from sporadic cases and taking into account that FH-II is the most common form of hereditary PA (6), the most recent Endocrinology PA guidelines recommend screening for familial FH type II in all hypertensive patients who have relatives with FH (66). However, we are aware that some patients classified as FH-II may be sporadic cases coexisting within the same family since until the discovery of the underlying genetic cause of FH-II, all cases with two or more positive cases of primary aldosteronism in the same family were classified as FH type II.

Type III FH is a severe form of PA characterized by extensive adrenocortical hyperplasia and hybrid steroid synthesis (42). The genetic defect is located in the *KCNJ5* gene. Women are disproportionately afflicted, accounting for more than 75% of all cases described in the literature. A higher prevalence of women has also been described in those sporadic PA patients who harbor a somatic pathogenic variant in *KCNJ5* (67). As we found, FH-III is frequently detected at a very young age and PAC reached double values than in sporadic cases. In addition, serum potassium levels are typically low, with a prevalence of hypokalemia nearing 90%. In fact, over 60% of these patients required a bilateral adrenalectomy to achieve adequate blood pressure control. Nevertheless, the severity of the PA varies depending on the *KCNJ5* pathogenic variant; for example, p.T158A, p.I157S, p.E145Q, and p.G151R are associated with early-onset severe PA (68) whereas p.G151E and p.Y152C present with mild PA (50, 59).

Only few cases of FH-IV have been reported in the literature (43). FH-IV is caused by germline defects in *CACNA1H*. Type IV, like FH-III, manifests at a very young age but later than the FH III cases. When compared with sporadic cases, serum potassium levels are also lower, but the degree of decline seems to be less severe than in FH type III. Patients with a family history of FH and early diagnosis of PA should be screened for familial FH type IV. Thus, the indications are identical to those for FH-III (45). In fact, if there is a suspicion of genetic PA, it is recommended that all genes be tested.

We are aware that our study has some limitations. The main limitation is that the definition of sporadic in the control group of sporadic patients was based on the epidemiological and clinical characteristics since the majority of patients in the SPAIN-ALDO did not undergo genetic testing. Nevertheless, cases with any suspicion of familial origin were excluded. Second, we have included all the cases reported in the literature following our strategy research that have available clinical and hormonal data, including patients from all over the world. Thus, we know that ethnic differences may also play a role in the many clinical and hormonal phenotypes detected. Thus, specific studies comparing sporadic and familial cases of the same ethnicity should be conducted. However, despite these limitations, this is the largest study comparing all the patients with FH of different types as well as sporadic PA cases.

## 5 Conclusion

In addition to being younger and having a family history of PA, FH-I and III share other typical characteristics. In this regard, FH-I is characterized by a low prevalence of hypokalemia and FH-III by a severe aldosterone excess causing hypokalemia in more than 85% of patients. The clinical and hormonal phenotype of types II and IV is similar to the sporadic cases. However, all genes should be tested if there is a possibility of genetic PA.

## Data availability statement

The raw data supporting the conclusions of this article will be made available by the authors, without undue reservation.

## Ethics statement

The studies involving humans were approved by CEIm Hospital Ramón y Cajal, Madrid. The studies were conducted in accordance with the local legislation and institutional requirements. The ethics committee/institutional review board waived the requirement of written informed consent for participation from the participants or the participants’ legal guardians/next of kin because Retrospective nature of the study.

## Author contributions

MA: Conceptualization, Data curation, Formal analysis, Investigation, Methodology, Writing – original draft, Writing – review & editing. PP: Writing – review & editing. PR: Writing – review & editing. MF: Writing – review & editing. MB: Writing – review & editing. EP: Writing – review & editing. AC: Writing – review & editing. JR: Writing – review & editing. AD: Writing – review & editing. EH: Writing – review & editing. RF: Writing – review & editing. IS: Writing – review & editing. MS: Writing – review & editing. RM: Writing – review & editing. MC: Writing – review & editing. PG: Writing – review & editing. CP: Writing – review & editing. LM: Writing – review & editing. RC: Writing – review & editing. ÁR: Writing – review & editing. PG: Writing – review & editing. CL: Writing – review & editing. MM: Writing – review & editing. MC: Writing – review & editing. SC: Writing – review & editing. DM: Writing – review & editing. MN: Writing – review & editing. VQ: Writing – review & editing. ER: Writing – review & editing. AS: Writing – review & editing. CD: Writing – review & editing. CO: Writing – review & editing. MT: Writing – review & editing. JG: Writing – review & editing. TM: Writing – review & editing. EM: Writing – review & editing. FH: Writing – review & editing.
